# Asphyxia, or the Appearances of the Teeth of Those Who Have Died from Strangulation

**Published:** 1841

**Authors:** 


### ASPHYXIA,

*Or the appearances of the Teeth of those who have died from strangulation.*

The following is an extract of a letter addressed to Professor Dungleson, at his request, by H. H. Hayden, M. D., of Baltimore, and published in the Encyclopædia of Medicine. It contains some very curious and interesting facts, and is interesting not only in a physiological point of view, but may be highly serviceable in medical jurisprudence.

"In certain varieties of asphyxia, the appearances of the teeth are not only singular, but highly instructive, in a physiological point of view, especially. They are almost uniformly tinged red. If examined immediately after death, they present a deep pink hue; if some time after, the tint is darker. The different shades of colour, however, will generally depend in a great measure, upon the age of the person, and the violence of the death:—for instance, the bony structure of the teeth of young persons is far more transparent and vascular than those of persons advanced in years. These appearances are particularly observable in the teeth of such as have been drowned, and more especially in those that have been hanged. They are, moreover, met with in the teeth of refractory bullocks that have been forcibly drawn into the slaughterhouse, by means of a rope round the neck. My attention was attracted to these and to many other appearances of the teeth about the year 1815; since which time I have observed them in numerous instances, both in human teeth and in those of the bullock.

On examining the teeth for the purpose of ascertaining, if possible, the nature and cause of these appearances, I found, on splitting them open through the roots, or fangs (which ought to be done from the edge of the crown, so as not to touch or disturb the vessels of the tooth,) the whole nervous pulp turgid, almost black, and even surrounded with blood. From this condition of the vessels, one might be led to suppose that the red tint of the teeth was reflected through the bone. But on removing the nerves, I found that the bony substance of the tooth was literally injected with the colouring matter of the blood, and this so effectually as to render every effort to restore it to its natural whiteness, perfectly ineffectual.

In this condition of things, my attention was directed to an examination into the causes of these phenomena, and the following, whether correct or not, is the result at which I arrived. In order perfectly to comprehend the process, it seems necessary to premise, that the nerves, arteries, and veins, which are destined to the teeth, both of man and animals, enter them through a minute orifice or foramen at the extremity of each root, and pass into a bony canal, which enlarges until it terminates in a cavity in the crowns of the teeth. Now, in the act of drowning, I consider that the heart continues to pulsate after the lungs have ceased to play; consequently, the blood is propelled to the head until the vessels are distended, and often ruptured, as is frequently the case with divers who remain long under water, the blood bursting from the eyes, nose, ears, and even from the mouth. In death, by hanging, or sudden strangulation, as in the case of bullocks, the truth of this position is corroborated by the following facts, which, in my mind, put the question beyond the reach of doubt. So soon as the drop falls, and the body is suspended from the gallows, or otherwise, respiration of course ceases. Still the heart continues, though irregularly, its action, the very first effort of which, after the return of blood, by the compression of the jugulars, is checked, is to force the blood into the arteries of the head, and consequently, into those of the teeth. The result is, that the arteries passing through the minute and unyielding foramina of the roots of the teeth, are so dilated, that the nerves and veins are compressed to such a degree that no blood can return by the latter. In the subsequent effort, the vessels are completely injected; and, ultimately, not only the vessels of the teeth are injected, and ruptured, but the bony substance of the teeth, the hardest and most compact in the human body, is penetrated by at least the colouring matter of the blood.

In a letter received from my much respected friend and professional confrere, the late Edward Hudson, Esq., dated Philadelphia, Sept. 11, 1818, he observes:—"In the small box you will find eight very fine teeth if they were not red. I want your opinion of what occasions this redness, premising that the teeth of all drowned persons are so, that I have examined."

In my reply, dated Sept. 26, 1818, I gave him a full explanation of the cause of those appearances, not only in the teeth of drowned persons, but also in the teeth of persons that have been hanged, and in those of bullocks drawn to the slaughter by a rope round the neck, &c., and asked him if I should return the teeth to him. In his letter and answer to me, dated Philadelphia, Jan. 31, 1819, he observes—"I take the present occasion to assure you, that you are very welcome to the teeth I was so fortunate as to send you. They are of use to you, and I have got some useful knowledge by a mere accident."

In 1833, I believe, two coloured persons were executed in this city, the one a male, the other a female; the latter for poisoning the wife of a medical gentleman. These subjects were examined by myself and two other medical gentlemen, after the heads had been in maceration seven weeks. The red appearance was strikingly obvious in all the teeth that were examined.

In the case of a coloured man who was executed in this city, last spring, for the murder of Capt. ——, these appearances of the teeth were equally evinced, and were, on that account, as I was informed, exhibited by Dr. Wm. Baker to his anatomical class."

